# The Role of the Gastrointestinal System in Neuroinvasion by SARS-CoV-2

**DOI:** 10.3389/fnins.2021.694446

**Published:** 2021-07-02

**Authors:** Jiali Xu, Zifeng Wu, Mi Zhang, Shijiang Liu, Ling Zhou, Chun Yang, Cunming Liu

**Affiliations:** ^1^Department of Anesthesiology and Perioperative Medicine, The First Affiliated Hospital of Nanjing Medical University, Nanjing, China; ^2^Department of Anesthesiology, Tongji Hospital, Tongji Medical College, Huazhong University of Science and Technology, Wuhan, China

**Keywords:** SARS-CoV-2, neuroinvasion, gut microbiota, gut- brain axis, gastrointestinal system

## Abstract

Coronavirus disease 2019 (COVID-19), caused by severe acute respiratory syndrome coronavirus 2 (SARS-CoV-2), is one of the most devastating pandemics in history. SARS-CoV-2 has infected more than 100 million people worldwide, leading to more than 3.5 million deaths. Initially, the clinical symptoms of SARS-CoV-2 infection were thought to be restricted to the respiratory system. However, further studies have revealed that SARS-CoV-2 can also afflict multiple other organs, including the gastrointestinal tract and central nervous system. The number of gastrointestinal and neurological manifestations after SARS-CoV-2 infection has been rapidly increasing. Most importantly, patients infected with SARS-CoV-2 often exhibit comorbid symptoms in the gastrointestinal and neurological systems. This review aims to explore the pathophysiological mechanisms of neuroinvasion by SARS-CoV-2. SARS-CoV-2 may affect the nervous system by invading the gastrointestinal system. We hope that this review can provide novel ideas for the clinical treatment of the neurological symptoms of SARS-CoV-2 infection and references for developing prevention and treatment strategies.

## Introduction

The World Health Organization (WHO) declared a coronavirus disease a public health emergency on January 30, 2020, and named it novel coronavirus 2019 (2019-nCOVID or COVID-19) on February 11, 2020 (Jin Y. et al., 2020; Zhu et al., 2020). Coronavirus disease 2019 is caused by an RNA coronavirus that belongs to the same genus *Betacoronavirus* as the previously identified severe acute respiratory syndrome (SARS) and the Middle East respiratory syndrome (MERS). Although COVID-19 has a lower mortality rate than SARS and MERS, it has a longer transmission cycle and is already infectious during the incubation period (Peiris et al., 2004; Zaki et al., 2012; Zumla et al., 2015; Astuti and Ysrafil, 2020; Li Z. et al., 2020).

Coronaviruses, initially isolated from poultry in the early 20th century, infect birds and mammals by targeting their respiratory and gastrointestinal mucosa (Wevers and van der Hoek, 2009). Mild or self-limited manifestations of respiratory disease are considered the major symptoms of human coronavirus infections. Coronaviruses can cause not only severe infections in the lower respiratory tract but also damages to the nerve, lung, liver, kidney, and other tissue and organs (Liu et al., 2020).

It is also common for coronaviruses to invade the nervous system. The viral RNA, such as that of HCoV-OC43 and 229E, can be found in human brain autopsy; both are common human coronaviruses. Moreover, neurological symptoms, such as acute disseminated encephalomyelitis and peripheral nerve diseases, have been found in patients with SARS and MERS (Montalvan et al., 2020). More than one-third of the laboratory-confirmed cases display symptoms such as dizziness, headache, myalgia, dysgeusia, dysosmia, and other signs of nerve involvement. In addition, critical ill patients and patients with comorbidities even exhibit symptoms such as delirium, acute vascular disease, and epilepsy (Heneka et al., 2020).

Although the nervous system is compromised by severe acute respiratory syndrome coronavirus 2 (SARS-CoV-2) infection, the underlying mechanism has not been elucidated. Based on the current literature and available case reports, there may be several routes for SARS-CoV-2 neuroinvasion: (1) invasion of the central nervous system after peripheral nerve infection, (2) direct involvement of the central nervous system through the olfactory bulb, (3) destruction of the blood–brain barrier (BBB) through lymphatic and hematogenous pathways, (4) severe destruction of the BBB caused by inflammatory responses to the virus, and (5) damage to the nervous system through the gut–brain axis (DosSantos et al., 2020; Kumar et al., 2020; Reza-Zaldivar et al., 2020; Dolatshahi et al., 2021). Because of the tropism of coronavirus to the gastrointestinal mucosa and the clinical manifestation of gastrointestinal diseases in patients with COVID-19, the gut–brain axis likely plays a vital role in SARS-CoV-2 neuroinvasion and comprehends an insight into its possible mechanisms.

## The Structure of SARS-CoV-2

Similar to other coronaviruses, SARS-CoV-2 produces 16 non-structural proteins and 4 structural proteins, including the membrane (M) protein, envelope (E) protein, spike (S) protein, and nucleocapsid (N) protein (Wang et al., 2020). The S proteins, which are essential for the recognition and invasion of host cells, form homotrimers protruding from the surface of the coronavirus. An S protein consists of two functional subunits, S1 and S2 (Wrapp et al., 2020). Most of the coronavirus S1 subunits possess receptor-binding domains (RBDs) that can specifically recognize host cell surface receptors and exhibit histotrophic properties according to the expression of the receptor gene (Li, 2016). When the S protein binds to the host cell surface receptor, the S2 subunit mainly mediates the fusion of the virus to the cell membrane (Naqvi et al., 2020).

Both SARS-CoV-2 and SARS-CoV can recognize and bind to the angiotensin-converting enzyme 2 (ACE2) on the cell surface. However, the subtle differences in the S protein between the two viruses result in a stronger affinity of SARS-CoV-2 to ACE2 (Li et al., 2003; Sternberg and Naujokat, 2020). According to The Human Protein Atlas, *ACE2* is highly expressed in the gastrointestinal tract, including the small intestine and colon, as well as the lungs (Li M. Y. et al., 2020). The exudation of inflammatory fluid and swelling of lung tissue lead to the fatal respiratory distress syndrome, caused by cytokine storm attributed to the abnormal release of pro-inflammatory cytokines (Jamilloux et al., 2020).

In patients with COVID-19, significant plasma cell and lymphocyte infiltration, interstitial edema, and even esophageal bleeding and ulcer erosion were observed in the gastrointestinal tract, suggesting that the gastrointestinal tract expressing *ACE2* was also the target of viral invasion (Zhang et al., 2020). The disruption of the intestinal immune balance further triggers the release of pro-inflammatory cytokines, expanding the state of excessive inflammation and eventually leading to death (Jin X. et al., 2020).

## The Symptoms of COVID-19

The symptoms of SARS-CoV-2 infection, similar to those of MERS-CoV and SARS-CoV infection, are usually characterized by unremitting fever, hypoxemic respiratory failure, systemic complications, encephalopathy, and thrombotic events (Su et al., 2016; Abboud et al., 2020; Najjar et al., 2020). The most common symptoms are respiratory, ranging from fever or mild cough to pneumonia, and involve multiple organ functions, ultimately leading to death with a mortality rate of 2%–4% (Wu et al., 2020; Xu et al., 2020).

In addition, most patients have noticeable gastrointestinal symptoms, such as nausea, vomiting, anorexia, abdominal pain, and diarrhea; some patients even only display gastrointestinal symptoms (Ahlawat et al., 2020). A study of 214 patients with SARS-CoV-2 found that 36.4% showed neurological symptoms, ranging from non-specific symptoms, such as dizziness, headache, and epilepsy, to more specific symptoms, such as acute encephalopathy (Mao et al., 2020).

Moreover, many patients with SARS-CoV-2 show global brain dysfunction; decreased alertness and awareness, often accompanied by overactive confusion and restlessness; or severe psychomotor retardation, accompanied by symptoms of malaise; it even quickly develops into a persistent coma (Najjar et al., 2020). A case report showed a 3-month-old girl developing non-febrile recurrent seizures after contracting SARS-CoV-2 in the COVID-19 family environment (Garcia-Howard et al., 2020). The patient had a cough and mild gastrointestinal symptoms before the onset of non-febrile seizures (Garcia-Howard et al., 2020).

## SARS-CoV-2 and Nervous System Diseases

Coronavirus disease 2019 (COVID-19) is often accompanied by neurological complications, especially in individuals with serious and critical diseases (Najjar et al., 2020). The neuroinvasiveness of SARS-CoV-2 has attracted much attention (DosSantos et al., 2020; Kumar et al., 2020). In a study of 33 autopsies of patients with COVID-19, SARS-CoV-2 RNA was found in the respiratory and cardiovascular regulatory center of the medulla oblongata (Meinhardt et al., 2021). In addition, a series of symptoms were observed in patients with COVID-19, including a broad range of neurological complications such as headache, ischemic stroke, disturbance of consciousness, and encephalitis/meningitis, indicating that SARS-CoV-2 could invade the brain (DosSantos et al., 2020; Meinhardt et al., 2021). In addition, in many case reports, other coronaviruses of the *Betacoronavirus* genus, such as SARS-CoV-1 and MERS-CoV, exhibited a neuroinvasive and neurovirulent profile (Chaves Andrade et al., 2020; DosSantos et al., 2020).

The significantly high homology between SARS-CoV and SARS-CoV-2 also supports the potential of neural invasion by SARS-CoV-2 (Reza-Zaldivar et al., 2020; Meinhardt et al., 2021). Neuroinvasion may occur through the retrograde synaptic transmission of SARS-CoV-2 from the mechanical and chemical receptors of the lung to the medullary cardiopulmonary respiratory center (Li Y. C. et al., 2020). In addition, *ACE2* is expressed by glial cells, neurons, and neurovascular endothelial cells in the brain. ACE2, as a functional receptor of SARS-CoV-2 for its entry, can bind to the viral surface spike glycoprotein (S1) and mediate SARS-CoV-2 into neurovascular endothelial cells (Baig et al., 2020; Najjar et al., 2020). The binding of ACE2 by the virus may decrease the activity of ACE2, further aggravating the neurological symptoms (Dolatshahi et al., 2021).

However, in most cases of severe neurological complications involving SARS-CoV-2, the reverse transcription polymerase chain reaction detection of SARS-CoV-2 in the cerebrospinal fluid samples was negative (Helms et al., 2020). This finding suggests that most of the neurological complications associated with SARS-CoV-2 may not be related to the direct entry of the virus into the central nervous system; instead, the complications may be caused by the increase in pro-inflammatory cytokines, resulting in abnormalities in the innate immune signal pathways in the central nervous system and a high peripheral inflammatory response (Dantzer, 2018). Therefore, the entry of pro-inflammatory cytokines into the central nervous system can activate glial cells and change their functions, leading to microglial activation and proliferation and further destroying the functional and structural integrity of the BBB (Dantzer, 2018). In addition, Cristian Follmer speculated that SARS-CoV-2 infection played an important role in the pathogenesis of neurological diseases, especially chronic degenerative neurological diseases such as Parkinson’s disease (PD). However, there is no evidence of an increase in the incidence of PD or any chronic neurological disease after infection with SARS-CoV-2 (Follmer, 2020).

## SARS-CoV-2 and Gastrointestinal Diseases

Reports of gastrointestinal symptoms in patients with COVID-19 have also risen along with the increase in the confirmed cases of COVID-19. The most common gastrointestinal symptom is diarrhea (11.5%), followed by nausea and vomiting (6.3%) and abdominal pain (2.3%) (Kopel et al., 2020; Silva et al., 2020). A considerable number of patients can still expel the virus through the gastrointestinal tract after their extra-gastrointestinal symptoms have disappeared (Kopel et al., 2020). In an observational study, eight patients developed diarrhea and fever before respiratory symptoms appeared. Thus, the first symptoms of COVID-19 may be gastrointestinal (Leung et al., 2003).

Currently, the most common route of SARS-CoV-2 transmission is through the respiratory tract. However, the detection of SARS-CoV-2 RNA in the feces and digestive tract also indicates that the virus invades the intestinal tract and may undergo fecal–oral transmission (Kopel et al., 2020). Since the epithelial cells of the small intestine also express *ACE2*, we believe that ACE2 is the gateway for SARS-CoV-2 to enter the intestinal cells. Severe acute respiratory syndrome coronavirus 2 can bind to ACE2 and infect the cells through the S protein, leading to gastrointestinal symptoms (Villapol, 2020). It has been widely recognized that gastrointestinal symptoms are usually accompanied by inflammation or gastrointestinal damage (Belkaid and Harrison, 2017; Villapol, 2020). Once the integrity of the intestinal barrier is damaged, intestinal microorganisms can activate innate and adaptive immune cells and release pro-inflammatory cytokines into the circulatory system, finally leading to systemic inflammation (Villapol, 2020).

## Relationship and Pathways Between the Intestinal Tract and Diseases of the Central Nervous System: Gut–Brain Axis

There is mutual communication between the intestinal and central nervous systems through various pathways (Cussotto et al., 2018). Enteric activity is not only involved in regulating the function of the central nervous system and influencing the behaviors of the host but also closely related to neuroinflammatory and neurodegenerative diseases (Foster et al., 2017). The vagus nerve, located in all the layers of the gastrointestinal wall, receives indirect stimulation from intestinal metabolites and bacterial compounds and integrates information to the central nervous system to maintain the balance between the brain and the intestinal tract (Bonaz et al., 2018).

The colonization of the mammalian intestine by enteric floras has important implications for the gut–brain axis. Bacterial metabolites, short-chain fatty acids (SCFAs), and lipopolysaccharide can directly act on the afferent fibers of the vagus nerve, which express toll-like receptors and neurotransmitter receptors at the end of the fibers. After receiving the stimuli, the fibers transmit the signals upward to the brain, thereby regulating intestinal immunity and permeability (Bonaz et al., 2017). Food components, such as carbohydrates, proteins, and fats, in the intestinal lumen also stimulate the endocrine cells (EECs) in the small intestine to produce intestinal hormones, 5-hydroxytryptamine (5-HT) and cholecystokinin (CCK). These hormones bind to receptors on the afferent fibers of the vagus nerve to regulate intestinal movement (Gunawardene et al., 2011).

Interestingly, EECs can also express toll-like receptors and bacteria-sensing products and stimulate the vagus nerve by secreting various hormones (Abreu et al., 2005). Microbiota transplantation is known to improve anxiety or depression-like behaviors in mice. However, no improvement was observed in mice undergoing vagectomy, suggesting that the microbiome and its metabolites and the vagus nerve play an integral role in the communication between the gut and the brain (Bravo et al., 2011).

In addition, cell proliferation and differentiation in the central nervous system are regulated by insulin-like growth factor-1 (IGF-1). Neuronal formation in the brain of most mammals stops after birth, whereas the neurons in the hippocampal dentate gyrus and subventricular areas are regenerated by IGF-1 induction (Anderson et al., 2002). The levels of circulating IGF-1 are reduced in germ-free mice compared with that in mice with normal intestinal flora, and the serum IGF-1 levels are increased after the transplantation of normal intestinal flora (Yan and Charles, 2018). Moreover, IGF-1 receptors are present in the intestinal mucosa, and IGF-1 is involved in the growth and metabolism of the intestinal mucosa. Reductions in intestinal mucosa atrophy were observed in mice with severe burn injuries treated with IGF-1 (Huang et al., 1993). This finding suggests that IGF-1 can inhibit inflammatory factors and intestinal bacteria from crossing the intestinal barrier and entering the blood and lymph circulation, thus reducing the damage to the BBB by suppressing systemic inflammation and the occurrence of brain diseases. Interestingly, there is also an interaction between the intestine and the lungs. Similar to the gut–brain axis, the gut–lung axis is thought to be bidirectional, and the intestinal flora can regulate the immunity of the lungs after viral infection. The significant difference between the gut–lung axis and the gut–brain axis is that intestinal bacteria can directly migrate to the lungs but only indirectly in the gut–brain axis (Conte and Toraldo, 2020; Dhar and Mohanty, 2020; Zhang et al., 2021).

## Pathways of SARS-CoV-2 Invading the Intestines: Inflammatory Response

The underlying pathophysiological mechanism underlying SARS-CoV-2 infection is complex and multifactorial; it has been summarized as the overaction of the immune system, which originated from inflammation/cytokine storm (Ferreira et al., 2020). Intact intestinal epithelial cells and the tight junction between cells constitute a mechanical barrier of the intestinal mucosa that can block the intestinal flora and resist external injury by regulating intestinal permeability (de Punder and Pruimboom, 2015). SARS-CoV-2 enters host cells through the ACE2 produced in the intestinal epithelium and downregulates the level of ACE2 on the cell surface (Iwata-Yoshikawa et al., 2019).

In the renin–angiotensin system (RAS), ACE2 mainly cleaves angiotensin (Ang) into Ang (1–7) that bind to Mas receptors and activate anti-inflammatory and anti-fibrosis pathways *in vivo* (Swirski et al., 2009; Sanchis-Gomar et al., 2020). SARS-CoV-2 interacts with resident lymphocytes in the intestinal epithelium and lamina propria to activate the host immune system (Azkur et al., 2020). The activation of intestinal immune cells and the imbalance of RAS-ACE2 result in the phosphorylation of p38 mitogen kinase and nuclear factor-κB pathways, producing inflammatory factors such as tumor necrosis factor α, interleukin-1, interleukin-6, and interleukin-8 (Beacon et al., 2020). As a result, the shedding and apoptosis of intestinal epithelial cells are induced, ultimately increasing the permeability of the intestinal mucosa.

The normal flora is also involved in forming the intestinal barrier, decomposing and providing nutrients to the intestinal mucosa and defending against viruses and bacteria (Diaz Heijtz et al., 2011). The apoptosis of the epithelial cells increases the permeability of the intestinal mucosa. Inflammatory factors, bacterial toxins, and intestinal pathogens can enter the blood and lymph circulation through the damaged intestinal barrier, further damaging the immune response of the host and resulting in a long-term state of systemic inflammation (Ferreira et al., 2020). Meanwhile, the tight junction proteins of the BBB can also be destroyed, allowing harmful substances to enter the brain and causing central nervous system lesions in patients with COVID-19 (Daneman and Prat, 2015).

## Abnormal Amino Acid Absorption

SARS-CoV-2 binds to the extracellular enzyme domain of ACE2 via the RBD of the S protein, and attaches to the surface of a host cell to enter the target cell for replication (Heurich et al., 2014). The interaction between the S protein and transmembrane serine protease 2 (TMPRSS2) on the cell surface activates the S protein, increasing the invasion of SARS-CoV-2 and induces the internalization and down-regulation of ACE2 through its cleavage after the virus entry (Zipeto et al., 2020).

ACE2 serves as functional peptidase and catalyzes the cleavage of Ang II in the RAS system; however, it also forms a complex with the amino acid transporter B^0^AT1 (*SLC6A19*) to mediate the absorption of neutral amino acids, such as valine, threonine, tyrosine, and tryptophan, in the enteric canal (Kuba et al., 2010). Tryptophan absorption via the ACE2–B^0^AT1 complex is significant in regulating immune, intestinal microorganism homeostasis, and susceptibility to enteritis (Segal et al., 1986). Compared with the wild-type mice, *Ace2* mutant mice produced fewer antimicrobial peptides in the gut, resulting in an imbalance in intestinal microbial composition (Salzman et al., 2010; Hashimoto et al., 2012). Dextran sulfate sodium (DSS) can destroy the intestinal epithelial barrier in mice and cause colitis. The supplementation of nicotinamide or tryptophan in *Ace2* mutant mice can improve the symptoms of DSS-induced colitis and diarrhea and promote the production of antimicrobial peptides in intestinal epithelial cells (Hashimoto et al., 2012).

In addition, tryptophan metabolites bind to aromatic hydrocarbon receptors (AHRs) on the surface of immune and epithelial cells; the activation of AHRs promotes the differentiation of Th17 or Treg cells. Indoles produced by tryptophan metabolism can traverse the BBB and combine with AHR in astrocytes to suppress pro-inflammatory activity (Gao et al., 2018). Feeding mice with a tryptophan-deficient diet aggravates the pathological changes in the central nervous system in a mouse model of experimental autoimmune encephalomyelitis. Consistent with these results, patients with multiple sclerosis also have an impaired uptake of tryptophan and its metabolites as anti-inflammatory mediators (Rothhammer et al., 2016).

## Intestinal Flora Imbalance

The imbalance of the intestinal flora in the gastrointestinal tract may also cause symptoms of the central nervous system (Foster and McVey Neufeld, 2013). Infection with SARS-CoV-2 can change the intestinal microflora (Ahlawat et al., 2020). The content of *Streptococcus*, *Clostridium*, *Lactobacillus*, and *Bifidobacterium* in the intestinal flora of patients with SARS-CoV-2 is relatively high, while the content of *Bacteroidetes*, *Roseburia*, *Faecalibacterium*, *Coprococcus*, and *Parabacteroides* is low (Guo et al., 2021). Angiotensin-converting enzyme 2, the gateway for SARS-CoV-2 to enter host cells, is abundant in the brush border of the intestinal cells in the ileum. Thus, SARS-CoV-2 can directly invade the intestinal cells, leading to changes in the intestinal flora (Guo et al., 2021).

The human intestinal tract, the niche of a large number of intestinal microflora, is mainly dominated by *Firmicutes* and *Bacteroides*, which produce a variety of metabolites to maintain intestinal homeostasis (Foster and McVey Neufeld, 2013). Intestinal microflora can affect immune functions such as the production of acetate and butyrate and protection from respiratory virus infection; changes in the intestinal microflora may induce behavioral changes, leading to depression and confusion (Dinan et al., 2015; Dhar and Mohanty, 2020; Shinu et al., 2020).

Behavioral changes may be related to the absorption rate of tryptophan in the gastrointestinal tract and the production of 5-HT in the brain tissue (Ahlawat et al., 2020; Yu et al., 2020). The intestinal microflora or its metabolic end products stimulate the vagus nerve, which can directly or indirectly regulate the production of neurotransmitters (Galland, 2014). In addition, the concentration of 5-HT in the intestine is maintained by the intestinal chromaffin cells with tryptophan hydroxylase and stimulated by intestinal metabolites, such as SCFA and bile acid (Cao et al., 2020). Therefore, we propose that the decrease of tryptophan levels may decrease serotonin levels, causing abnormality in the gastrointestinal flora, finally leading to neurological symptoms (Shinu et al., 2020).

Because the imbalance in the intestinal flora can cause this series of changes, a single or mixed culture of practical dietary fiber and probiotics, including living microorganisms, can strengthen the immune function by maintaining the stability of the intestinal microflora (Conte and Toraldo, 2020; Dhar and Mohanty, 2020; Olaimat et al., 2020). In addition, probiotic intervention may improve the effectiveness of a vaccine, further preventing a virus infection (Karl, 2021).

## Retrograde Transport of Enteric Nerves

It has been observed that some patients with SARS-CoV-2 infection experience a loss of the sense of smell, suggesting that SARS-CoV-2 can penetrate the nervous system through the olfactory bulb through the TRPMSS2 receptor (Dolatshahi et al., 2021). After the SARS-CoV-2 invades the gastrointestinal system, it can also reach the central nervous system through direct neuroinvasion or indirect immune activation of the intestinal nervous system or through the afferents of the intestinal vagus nerve (Bostanciklioglu, 2020). Chaves Andrade et al. hypothesized for the first time that the symptoms initially appearing in the nervous system, such as headaches, disturbance of consciousness, and sensory abnormalities, might be subjective; however, more serious symptoms, such as disturbance of consciousness, seizures, and strokes, might occur during infection and inflammation (Chaves Andrade et al., 2020). Studies have shown that glial cells and neurons in the central nervous system can express ACE2, which mediates the entry of viruses into glial cells, resulting in neurological symptoms (Belkaid and Harrison, 2017). Therefore, we propose that SARS-CoV-2 can retrogradely invade the glial cells of the central nervous system through the intestinal nervous system after invading the gastrointestinal system, causing neurological symptoms (Chaves Andrade et al., 2020). Thus, treatment measures, such as vagus nerve stimulation, may inhibit the neuroinvasion of COVID-19 (Chaves Andrade et al., 2020; Esposito et al., 2020).

## Conclusion

**FIGURE 1:**
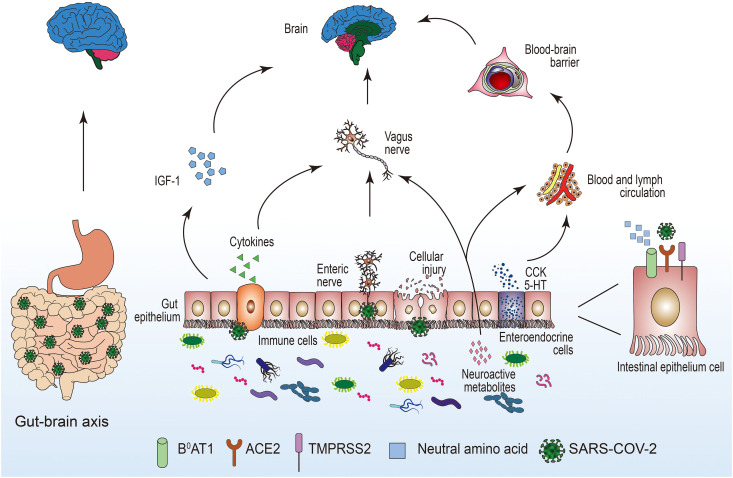
The potential pathways for the invasion of SARS-COV-2 to the central nervous system through the intestinal tract. 5-HT, 5-hydroxytryptamine; ACE2, angiotensin-converting enzyme 2; B^0^AT1, amino acid transporter; CCK, cholecystokinin; IGF-1, insulin-like growth factor-1; SARS-COV-2, severe acute respiratory syndrome coronavirus 2; TMPRSS2, transmembrane serine protease.

Coronavirus disease 2019 (COVID-19) is a pandemic caused by SARS-CoV-2; patients with COVID-19 often are accompanied by neurological complications. In this review, we mainly explored the pathophysiological mechanisms of neuroinvasion caused by SARS-CoV-2 through the gastrointestinal tract (Figure 1). The invasion of the nervous system by SARS-CoV-2 after infection has been confirmed. Therefore, we proposed that the gastrointestinal tract plays a critical role in the process of SARS-CoV-2 neuroinvasion. The gastrointestinal tract may be the portal for SARS-CoV-2, and SARS-CoV-2 may directly or indirectly invade the nervous system after many replications in the gastrointestinal tract. The early gastrointestinal symptoms after SARS-CoV-2 infection are usually mild, often characterized by diarrhea, and thus easy to overlook. We believe that the emergence of gastrointestinal symptoms, such as diarrhea, has more important clinical significance. Monitoring the emergence of gastrointestinal dysfunction is of great significance for the prevention and treatment of COVID-19; it can enable us to better understand the progress of COVID-19 to prevent the emergence of serious neurological complications after SARS-CoV-2 infection.

## Author Contributions

This manuscript was primarily written by JX and ZW. MZ and LZ collected the data in this review article. CY and CL provided guidance for the creation of this manuscript. SL and CL contributed to the critical feedback of the manuscript for crucial content. All authors contributed to the article and approved the final manuscript.

## Conflict of Interest

The authors declare that the research was conducted in the absence of any commercial or financial relationships that could be construed as a potential conflict of interest.
